# Employee loyalty evaluation using machine learning in technology-based small and medium-sized enterprises

**DOI:** 10.1038/s41598-025-06475-y

**Published:** 2025-07-02

**Authors:** Yong Shi, Yuan Wang, Hongkun zuo

**Affiliations:** 1 School of Computer Science, Huainan Normal University, Huainan City, Anhui China; 2 School of Finance and Mathematics, Huainan Normal University, Huainan City, Anhu China

**Keywords:** Information technology, Sustainability

## Abstract

Employee loyalty is a major issue of sustainable human resource management. Small and medium-sized enterprises with high technology content and strong innovation ability are the main body of innovation with great vitality and potential. Employee loyalty is an important factor for the success and development of Technology-based Small and Medium-sized Enterprises (TSMEs). This research starts with the historical evaluation data of employees in Chinese TSMEs to analyze the relevant factors of employee loyalty and the relationship between these factors and employee loyalty. Through several machine learning models and algorithms to predict employee loyalty, the feasibility of machine learning to predict employee loyalty is proved, and the evaluation of talent in TSMEs is supported by decision analysis. This research aims to build an objective and applicable intelligent evaluation model of employee loyalty to support TSMEs to accurately identify, motivate, attract, cultivate and retain outstanding scientific and technological talents. This study contributes to the promotion of sustainable and high-quality management of TSMEs.

## Introduction

Technology-based Small and Medium-sized Enterprises (TSMEs) refer to enterprises that rely on a certain number of scientific and technological personnel to engage in the development and application of products and new technologies in the fields of information, electronics, biological engineering, new materials, new energy and other technological industries, so as to achieve sustainable development. At present, there are more than 2 million scientific and technological enterprises in China. TSMEs with high technology content and strong innovation ability are the main body of innovation with great vitality and potential. Scientific and technological SMEs are an important force to strengthen the main position of enterprise innovation. Gathering high-end talents is the cornerstone of the development and growth of TSMEs. How to motivate, attract, discover, train and retain scientific and technological talents loyal to the enterprise is an important topic for the whole society and TSMEs.

Employee loyalty has an important impact on the operation and development of TSMEs, especially on the company’s core competitiveness, labor replacement cost and enterprise performance. Employee loyalty refers to the behavioral orientation and psychological connection of employees to the enterprise, that is, the degree of dedication of employees to the enterprise they serve. Employees stay with the company for a long time because they feel valued, appreciated, and believe in the overall mission of the company, which generates loyalty. Employees who are loyal to the company are likely to be more invested in the success of the company and work hard to achieve organizational goals.

Enterprises should understand the necessary behaviors and practical activities to improve employee viscosity and ensure that the team will voluntarily and systematically take responsibility and implement them very seriously. Employee loyalty and employee value evaluation have a direct impact on talent mining. Strenitzerov and Achimsk (2019) pointed out that the management of employee loyalty is a major issue of sustainable human resource management^1^.

Using modern information systems and human resource management technologies, it can provide operational assistance in collecting, storing and preparing report data, coordinating and optimizing process and data control, and providing timely information to enterprise managers^2^.In any organization, employee loyalty is an important factor in gaining competitive advantage. Research shows that employee loyalty is significantly related to corporate image and employee satisfaction.^3^.

Vasumathi (2021) believes that employee loyalty is the most important factor for an enterprise to achieve success and development and it is an important attribute for any organization to gain competitive advantage. Moreover, it will bring a lot of internalized productivity to companies and enhance the overall value of the enterprise^4^.

Studies show that in all sorts of different sample groups about how to improve employee loyalty and value of research has a long history, but many scholars are based on the perspective of big companies, such as some large state-owned enterprises and multinational companies, etc. Due to the uniqueness of TSMEs described earlier, it cannot be determined whether traditional methods are applicable. However, due to the large size, wide- coverage, relatively weak strength, and increasingly fierce competition, it is necessary to conduct theoretical research and practical exploration on employee loyalty in TSMEs. However, few studies have targeted TSMEs employee loyalty using machine learning. Based on the above literature, this research attempted to fill the deficiencies in the existing research. The human resources department of the enterprises being studied evaluates employee loyalty every year, thus accumulating the employee loyalty historical evaluation data. In terms of the specific forms and means of the information system used by the TSMEs to carry out work, only a certain degree of data analysis and statistics can be carried out, which cannot achieve the function of decision support. Therefore, for the TSMEs, the decision analysis of employee loyalty is bound to bring effective improvement to enterprise human resource management.

As a part of the application technology of human resource management, machine learning can help human resource managers break away from simple and repeated routine tasks in the process of playing its role, so that they can focus more time and attention on strategic human resource management and planning, organizational development, and other tasks, thus creating more value for the organization. Compared with traditional talent value analysis and management, the biggest advantage of machine learning lies in the formation of big data with different dimensions for each employee, which enables human resources departments to collect and count employee evaluation results more effectively, so as to provide a reference for managers’ decision support (Tong, 2021)^5^. This research can provide strong support and help for TSMEs in human resource management and enterprise management, and can promote the sustainable, healthy, and stable development of TSMEs to a certain extent. This research has certain theoretical value and practical significance in the development and management of human resources in modern enterprises.

The first is to establish significant predictors that predict employee loyalty, specifically improve employee loyalty, and value, and enhance the core competitiveness of TSMEs. This research can deeply explore the important indicators that affect the loyalty of employees, to improve the loyalty of employees and create more competitiveness for enterprises.

The second is to accurately predict the employee loyalty of the company, reduce the labor replacement cost of the enterprises, and promote the sustainable and healthy development of the company. If this research can predict the employee loyalty of the enterprises in time and manage human resources effectively, it will greatly improve the company’s harmony and stability and improve the company’s overall efficiency.

The third is to timely adjustment of employee management methods, identify talents and find talents, cultivate talents, and support the talent management of TSMEs. Through the evaluation research on the value of the company’s employees, after data mining and system development, the company proposes relevant countermeasures and suggestions to improve employee loyalty and promotes the sustainable, healthy, and stable development of the enterprises.

In summary, this article focuses on the following structural issues for research. What important predictive indicators are determined through machine learning algorithms to predict employee loyalty? What are the benefits of accurately predicting employee loyalty for a company? How to support talent management in TSMEs through employee value assessment research?

The acronym used in the rest of this paper is tabulated in Table 1.The specific machine learning algorithms will be explained in depth in the following text.

**Table 1:** Table 1 describes the acronyms.

Acronyms	Description
ML	Machine Learning
SMEs	Small and Medium-sized Enterprises
TSMEs	Technology-based Small and Medium-sized Enterprises
KNN	K-Nearest Neighbor
NB	Naive Bayes
NN	Neural Net
RF	Random Forest
LR	Logic Regression
SVM	Support Vector Machine
LR	Logistic Regression

## Related research

The following related studies provide good support for this research.

### Informationization management of enterprise human resources

Sharipov et al. (2020) pointed out that human resource management within a company is crucial for its success and development. In the digital economy, the use of information technology is crucial for knowledge management and decision-making processes^6^. Enterprise information systems also have a significant impact on key performance indicators in construction project management, affecting costs and profits (Mesároš et al., 2021)^7^. Furthermore, Liu et al. (2021) achieved optimized allocation management of human resources in enterprises by using adaptive neural network models and data mining control, thereby improving efficiency^8^. Ruan et al. (2021) concluded that government crisis management restored the confidence of managers through different mediators of sense of gain. Information and communication management will only enhance their sense of spiritual gain, while human resource development will only enhance their sense of material gain^9^. Chatbots are also being integrated into human resource management to provide real-time assistance and support, although challenges such as cost factors and limited responsiveness need to be addressed (Majumder et al., 2021)^10^. Additionally, the integration of blockchain technology in production scheduling and human resource management allows for the automation of selecting human resources for production tasks, enhancing efficiency and effectiveness (Balon et al., 2022)^11^. In conclusion, the effective management of human resources within an enterprise is essential for its success and growth. Utilizing information technology, big data mining, and innovative technologies such as blockchain can significantly improve human resource management processes, decision-making, and overall performance within organizations.

### Data mining of enterprise talents

The literature on value mining of enterprise talents encompasses various aspects of utilizing data mining techniques to enhance human resource management and talent development within organizations. Xu et al. (2021) propose a data mining method for enterprise human resource management based on the simulated annealing algorithm, resulting in improved nurse scheduling efficiency and satisfaction^12^. Ma (2021) explores the application of data mining and information technologies to address human resource management challenges within companies^13^. Huang et al. (2021) analyze the demand characteristics of wisdom supply chain management talents through data mining methods in the Yangtze River Delta region^14^. Fan (2021) discusses financial process reengineering and personnel transformation based on financial shared services^15^. Zhang (2021) established a human resource management system using single decision tree algorithms including C4.5, random tree, J48, and tested the evaluation management and talent recommendation modules^16^. Derevyanko et al. (2021) delve into strategic enterprise management based on economic security modeling^17^. Xie et al. (2022) study the measurement of the contribution value of scientific and technological talents in China’s mining industry using data analysis methods to determine their impact on national economic development^18^. Ye et al. investigated 225 employees in the context of enterprise digital transformation, and the results showed three driving paths to promote employee work engagement: open experience awareness, self-efficacy drive and inhibition of technology stressors^19^. These studies collectively highlight the significance of data mining and information technologies in enhancing talent management and organizational decision-making processes.

### Factors influencing talent loyalty in enterprises

Talent loyalty in enterprises is a crucial aspect that can significantly impact the success and sustainability of businesses. Oliver (2016) used the employee firm matching data of the Work and Employment Relations Survey (WERS) in 2011 to estimate ordinary least squares (OLS), instrumental variable (IV) model and treatment effect model and concluded that employee loyalty was negatively correlated with wages^20^. Hou (2020) analyzed the influencing factors of enterprise talent innovation, highlighting the importance of network relationships in improving innovation capacity^21^. Some studies pointed out that satisfaction and trust play a dual mediating role in the relationship between leadership support and loyalty (Aristana et al.,2021)^22^. Hyeon (2022) reveals that perceived ease of use has a significant impact on perceived usefulness and perceived enjoyment, and proposes that ICT skills have a positive impact on both loyalty and satisfaction^23^. Through in-depth analysis of the connotation, influencing factors and promotion strategies of employee loyalty. Dong (2024) provides valuable references and suggestions for SMEs, which is helpful for enterprises to improve employee loyalty^24^. Overall, understanding and addressing the factors influencing talent loyalty in enterprises are essential for fostering a positive work environment, promoting innovation, and retaining valuable employees. Further research in this area can provide valuable insights for businesses seeking to enhance talent loyalty and overall organizational success.

### Application of machine learning and data mining technology to evaluate employees

The application of machine learning and data mining technology in evaluating employees has gained significant attention in recent years. Gao et al. (2022) proposed a productivity prediction method for unconventional natural gas wells using artificial intelligence and data mining technologies^25^. Ruckmani et al. (2020) conducted a comparative study on software defect prediction using machine learning methods, highlighting the importance of developing effective error-finding models^26^. Andreea(2020) identifies a way to assess whether managers are promoting or providing employee benefits objectively^27^. In the study, three data mining algorithms were evaluated to determine their level of efficiency when applied to available data sets. Following the Data Mining Cross-Industry Standard Process (CRISP-DM) and Data Science Lifecycle process, Sumali et al. (2021) use machine learning techniques to analyze employee reviews. The goal is to predict an overall measure of employee satisfaction^28^. Pessach et al. used a uniquely large dataset containing recruitment records for hundreds of thousands of employees over more than a decade and representing a wide and heterogeneous population. The analysis shows that the VOBN model can provide highly accurate and interpretable insights for HR professionals^29^. Tanasescu et al. (2024) reduced the impact of human opinion on employee evaluations and improved objectivity and overall productivity by preprocessing data, selecting the best variables, building the best algorithms for the available data, and adjusting its hyperparameters^30^. Shi conducted machine learning research on the performance of human resource prediction algorithm models^31^. These technologies offer valuable insights and predictive capabilities that can enhance decision-making in various organizational contexts.

### Employee data mining technology

Employee data mining technology has been increasingly utilized in various industries to improve operational performance and decision-making processes. Qi (2020) discussed the basics of big data management in the mining industry and its potential benefits^32^. In the context of intelligent manufacturing, Guo et al. (2020) introduced an Internet of Things-based decision support system that incorporates data mining technology for information processing^33^. Goksoy (2021) explored the relationship between internal social media, data mining, and change management in Industry 4.0, highlighting the role of social media in facilitating change and data mining in providing insights into employee perceptions^34^. Ghazi et al. (2021) proposed a data mining framework to predict employee turnover among information technology specialists, focusing on attitudinal employee characteristics^35^. Yang et al. introduced a variety of database and data mining techniques to help clinical researchers better understand and apply database technologies^36^. Furthermore, Anaam et al. (2021) emphasized the importance of data mining techniques in electronic customer relationship management for the telecom industry to enhance decision-making quality^37^. Overall, the literature review demonstrates the diverse applications of employee data mining technology across different sectors, highlighting its significance in improving operational efficiency and decision-making processes.

### Machine learning classification model

Joseph et al. (2021) focused on predicting employee attrition rates and emotional assessment using algorithms such as Decision Tree Classifier, Support Vector Machine, and Random Forest Classifier^38^. Konnaris et al. (2021) compared two machine learning classification models for automated histomorphometry, showcasing the importance of selecting the appropriate model for specific applications^39^. In the context of skill-based labor market analysis, Mrsic et al. (2020) leveraged machine learning and big data to automate resume/skill classification and improve productivity. They proposed a model that extracts important information from resumes, classifies skills using ESCO, and utilizes interactive visualization tools for smart insights^40^. Perrot et al. (2019) investigated the accuracy of a machine-learning classifier in differentiating between kidney stones and phleboliths on low-dose computed tomography, achieving an overall accuracy of 85.1%^41^. Moreover, the study by Kang et al. (2021) focused on perceived organizational performance in U.S. federal government health agencies using a machine-learning classification decision-tree model. They identified several tree-splitting variables and classified employees into high-risk, moderate-risk, and low-risk subgroups^42^. Overall, these studies highlight the importance and effectiveness of machine learning models in predicting employee performance, attrition rates, organizational performance, and turnover.

By sorting out domestic and foreign research literature on employee loyalty, it can be found that in terms of research content, the existing achievements mainly focused on HRM information practice, employee loyalty evaluation and influencing factors exploration, application of machine learning in employee prediction and evaluation, algorithm comparison and selection, prediction model testing and analysis, and related development technology research.

In terms of research methods, most of the literature adopt a combination of theoretical and empirical research, following the structural steps of theoretical elaboration, research framework design, research and comparison of employee turnover prediction models, and data analysis and verification. In the selection and innovation of the algorithm, the main performance is to adjust the relevant parameters of the algorithm or the fusion of various algorithms to maximize the prediction accuracy.

## Methods

**Fig. 1:**
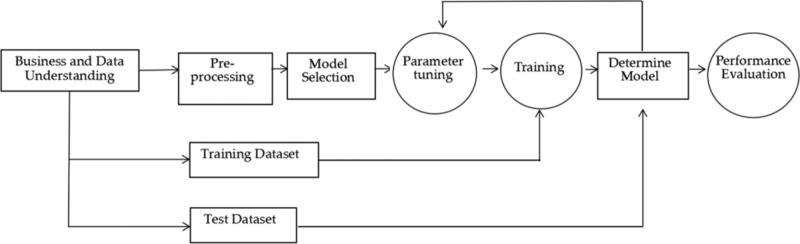
The process of model construction.

This study is conducted in an honest, transparent and ethical manner. It was approved by the ethics committee of Huainan Normal University. All methods of this study were carried out in accordance with relevant guidelines and regulations. All experimental protocols for this study have been approved by the designated institution. All participants provided informed consent. We obtained written consent from participant involved in the study. This informed consent ensures that their rights and privacy are protected throughout the research process. This research was conducted to better determine the loyalty of employees to the enterprises. This research needed to address the following concerns: the methods for analyzing and studying relevant historical data about employees; the ways to analyze and explore factors and models that influence employee loyalty. Figure 1 shows the model building process.

Business and Data Understanding: This part includes business understanding and business data understanding, employee management system, loyalty mining, value analysis and other business requirements and data. Data acquisition is a very important step in the data mining process as the quality of the collected data will directly affect the training effect of the model and the accuracy of the subsequent prediction.

**Data pre-processing:** It mainly includes data cleaning, data integration, data transformation and data specification.

**Data mining:** This step is employee loyalty mining analysis. Various dimensional models are used to mine data and analyze the relationship between data variables.

**Model evaluation:** This refers to the evaluation of the model performance. To evaluate the effect of the model and whether it can meet the business needs of human resource management, various evaluation methods and indicators should be applied, and even the managers of TSMEs should be involved to thoroughly evaluate the model. In this research, algorithm performance was evaluated. After the evaluation, if the evaluation passes, it will enter the deployment phase, otherwise, it will be iteratively updated again.

### Data sources

The first step of research is to collect and store data related to employee loyalty. This step is the operation layer of the original data, and the existing data storage forms of the enterprises are relatively diverse, which may be text files or relational databases.

The total number of technology enterprises in the Yangtze River Delta exceeds 200,000.The total number of science and technology talents in 41 cities in China’s Yangtze River Delta has gradually increased. With the development of the Yangtze River Delta regional integration as a national strategy, its competitiveness, fluidity, openness, sharing and other features become more and more obvious. The data in this research came from the employees and evaluation data of enterprises in the Yangtze River Delta region of China. Because these enterprises are the partners of the research unit, they provide a good practical basis for this research. These enterprises have been engaged in industrial software development and application for a long time, and have obtained many qualification certificates such as ISO full series system certification, CMMI software Maturity Level 3 certification, ITSS operation and maintenance System level 3 certification. These enterprises are committed to building a professional, stable, well-structured and dynamic team of highly skilled talents.

Through the survey and interview of partner enterprises, the research collected a number of data related to employee loyalty and formed the original data after integration. Firstly, the main data was obtained through direct interviews with employees. During the interview, we designed a series of questions around the theme of employee loyalty. Through these in-depth interviews, a solid foundation has been provided for the subsequent analysis of the factors influencing employee loyalty. Secondly, the data is obtained from the business systems of partner enterprises. The business system of partner companies records detailed information such as employee work performance, attendance, training records, project participation, and promotion records. Thirdly, the data obtained was also obtained from the official websites of partner companies. The official website of partner companies usually contains information about the company’s corporate culture, values, development history, honors and awards, as well as employee activities. We have conducted a systematic review and cleaning of company records to ensure the integrity and consistency of the data. The interview data is recorded in the form of audio recordings and notes, and organized and analyzed by professionals to extract key information. The data on the official website, such as employee evaluations and messages, has also been screened and verified to exclude false or misleading information.When integrating these data, we used cross validation to compare data from different sources and ensure their consistency. For the data with discrepancies, we conducted further investigation and verification to ensure that the final data used is accurate and reliable. Through this integration and verification process, we have laid a solid foundation for subsequent employee loyalty prediction and analysis.

The following is a detailed description of the interviewees. Senior Leaders are the supporters of the development of this system, and also the decision makers of the company, who can propose constructive suggestions for the whole process of the research. Head of Human Resource Management department are the main users of the system and the people who collect all kinds of evaluation and result data. Through them, more opinions on system construction can be obtained and they play an important role in data collection and system evaluation. Employees are one of the sources of system development data acquisition, at the same time, they are also direct testers, therefore, their feedback information strongly supports the development of the system. In addition, they play an important role in data collection. Department Leaders are the most direct personnel to mine the results of employee loyalty and value evaluation and obtain direct opinions through them. Lastly, the leaders were the ones responsible for verifying the collected data.

The main interview guide questions are listed below. What problems have you encountered in developing employee loyalty prediction? How do you usually carry out employee loyalty analysis? Do you think it is necessary to systematically manage employee loyalty prediction? What problems did you encounter in preparing the report materials? What factors do you think affect employee loyalty? Do you think the employee loyalty management system with decision support will play a significant role in the development of your company? If possible, would you encourage the company to use the employee loyalty management system with decision support as a part of human resource development?

Reliability analysis is used to evaluate the stability and consistency of a measurement tool or test. In interview analysis, reliability usually refers to the reliability and consistency of interview data. In this study, SPSS 25.0 statistical software was used to analyze the interviews, and it was concluded that Cronbach’s alpha coefficient was greater than 0.8, with good reliability.This indicates the internal consistency of multiple measurement items when measuring the concept or property in this study.Feedback method, comparison method and participant test were used to test and improve the validity of the interview. The above methods ensure the effective implementation of the interview.

Before data mining of employee loyalty, the collected data needed to be integrated to form raw data. After data collection and preliminary preprocessing, this research integrated 2200 pieces of data and Python language was used to analyze and mine the data of employee loyalty.

Data sources include Database and Datastore. This research communicated with the leaders of enterprises, who assigned relevant data specialists to coordinate the company’s data collection while data collection in this research was carried out by a combination of random sampling and stratified sampling. In order to avoid introducing bias into the research results, when selecting stratification criteria, full consideration should be given to the heterogeneity of the population and the research objectives, ensuring that each layer can fully represent different subgroups in the population. In addition, the impact of stratified sampling on research results was evaluated through methods such as cross validation to ensure the accuracy and reliability of the research findings. After determining the stratification criteria, employees are generally divided into different levels. According to the department, the employees are divided into administrative department, sales department, R&D department and production department. According to the ratio of the number of employees of each layer to the total number of employees and the total number of samples required for the research, the number of samples taken from each layer is calculated to ensure that the sum of the sample amounts taken from each layer is equal to the total number of samples. Within each layer, a specified number of employees are sampled using a random sampling method. In simple random sampling, the random number table method is used to ensure that each employee has an equal probability of being selected. Due to the developed economy and dense population in the Yangtze River Delta region, there are large economic and cultural differences between different cities and regions. Therefore, in the sampling process, full consideration should be given to the representativeness of these regions and industries to ensure that the sample can represent the overall characteristics of the entire Yangtze River Delta region. At the same time, the research covers multiple departments, such as production, research and development, sales, etc. When sampling, ensure the representation of different economic sectors to avoid sampling bias caused by sectoral differences. At the same time, strict quality control in the data collection process, such as training investigators and reviewing and cleaning the data, can reduce errors.

Moreover, the current study analyzed the loyalty of employees and based on the findings, positive samples were those with high loyalty while negative samples were those with low loyalty. Therefore, the data needed to be sampled from two levels in a balanced ratio. The specific sampling method was to randomly extract and stratify the original data from the company’s personnel management system and employee management system, and then clean, integrate and standardize the data after it is exported.

### Predictors

According to the current research status, there are many factors that affect employee loyalty. In specific industries and positions, there are different influencing factors.Collect relevant data of employees through previous questionnaire surveys, interviews, enterprise databases, and other methods. Determine the important factors based on the following considerations. The generation of predictors in this study is based on the following criteria and contents.

The first is relevance. The predictor should have a significant correlation with the forecast target. The method is to identify and select relevant fields through Pearson correlation coefficient correlation analysis.

The second is completeness. The data set contains enough information to train the model, that is, enough fields need to be selected to capture various features of the data.The method is to ensure the integrity of the data set through data preprocessing, and possibly add new relevant fields through feature construction, feature scaling, feature coding, etc.

The third is accuracy. The data for the predictor should be as accurate as possible to reduce the impact of noise and error on model training. Methods To improve the accuracy of data by removing duplicate data, correcting error data and transforming data.

The fourth is interpretability. In some cases, predictors are interpretable so that the model’s decision-making process can be explained when it makes its predictions.Research selects fields with clear enterprise or business implications and avoids using overly complex features or combinations of features. At the same time, interpretable machine learning models can be used to enhance the interpretability of the model.

Factors with low correlation, incomplete data, inaccurate data, and unclear descriptions have been filtered and removed. Ultimately, 17 factors were retained. Specifically, Age represents an employee’s physiological age; Gender refers to an employee’s biological sex; Education level reflects an employee’s knowledge background and academic attainment; Position indicates an employee’s role and responsibilities within the organization; Salary level reflects an employee’s financial compensation; vacation days concern an employee’s leave time; Welfare level represents the benefits an employee enjoys; Ability Level embodies an employee’s skills and competencies; Team Spirit reflects an employee’s cooperative attitude within a team; ambition denotes an employee’s career goals and aspirations; Value Recognition embodies an employee’s self-perception of their value; Honesty reflects an employee’s integrity; Belonging Sense indicates an employee’s loyalty and identification with the organization; Training Opportunities represent the likelihood of an employee receiving training; Promotion Opportunities reflect an employee’s prospects for advancement within the organization; Working Environment describes the physical and psychological conditions in which an employee works; Overtime involves an employee’s working hours and intensity. Nevertheless, the following text will conduct important analysis and correlation analysis on the existing 17 factors, as well as provide comments and descriptions, in order to more accurately predict the core important factors and their correlation discourse. Subsequently, this research adopted machine learning algorithms. Hence, there are 17 main predictors, as shown in Table 2 below.

**Table 2:** Main predictors. Table 2 is the Predictors table. As shown, there is a total of 16 predictors used in this research. These were used to predict employee loyalty.

Predictors	Description	Remark
Age	Employee Age	Specific figure
Gender	Employee Gender	Male, female
Education	Employee Education Level	junior, college, bachelor, master, doctorate
Position	Employee Job Position	low, medium, high
Salary	Employee Salary Level	Level 0–5 from low to high
Vacation	Employee Vacation Level	few, medium, many
Welfare	Employee Welfare Level	low, medium, high
Ability Level	Employee Ability Level	Level 0–5 from low to high
Team Spirit	Employee Team Spirit Level	Level 0–5 from low to high
Ambition	Employee Ambition	Level 0–5 from low to high
Value Recognition	Employee Value Recognition	Level 0–5 from low to high
Honesty	Employee Honesty	Level 0–5 from low to high
Belonging Sense	Employee Belonging Sense	Level 0–5 from low to high
Training Opportunities	Employee Training Opportunities Level	low, medium, high
Promotion Opportunities	Employee Promotion Opportunities Level	low, medium, high
Working Environment	Working Environment Level	low, medium, high
Overtime	Employee Overtime Level	low, medium, high

According to the table, predictors include Age,Gender, Education, Position, Salary, Vacation, Welfare, Ability Level, Team Spirit, Ambition, Value Recognition, Honesty, Belonging Sense, Training Opportunities, Promotion Opportunities, Working Environment, and Overtime.

### Data preprocessing

In data mining, there are a large number of incomplete, inconsistent and abnormal data in the original data, which seriously affects the execution efficiency of data mining modeling and may even lead to the deviation of mining results. Therefore, data cleaning is particularly important. After data cleaning is completed, a series of processing such as data integration, conversion and specification are carried out, which is data preprocessing. On the one hand, data preprocessing is to improve the quality of data. On the other hand, it is to make data better adapt to specific mining technologies or tools. In this paper, the main contents of data preprocessing include data cleaning, data integration, data transformation and data specification. After data cleaning and preprocessing, the data is stored in the database or database warehouse for the use of data mining at the next level.

This research first deals with missing values and abnormal data. Missing value refers to clustering, grouping, deletion, or truncation of data due to lack of information in rough data. Missing values occur because the values of one or some attributes in the existing dataset are incomplete. The methods to deal with missing values generally include deletion and interpolation of missing values. For high-dimensional data, the noise features can be removed to reduce the interference to the model. Interpolation methods include nearest neighbor interpolation, mean interpolation, median filling and other methods. In this research, the interpolation method and mean value method before and after interpolation are comprehensively used.

Abnormal data is a special case that is often encountered in data analysis and the so-called abnormal value is abnormal data. Sometimes abnormal data is not only useless but will also affect the normal analysis results. Therefore, in the process of data exploration, it is necessary to identify these abnormal data and be handled well.

**Fig. 2:**
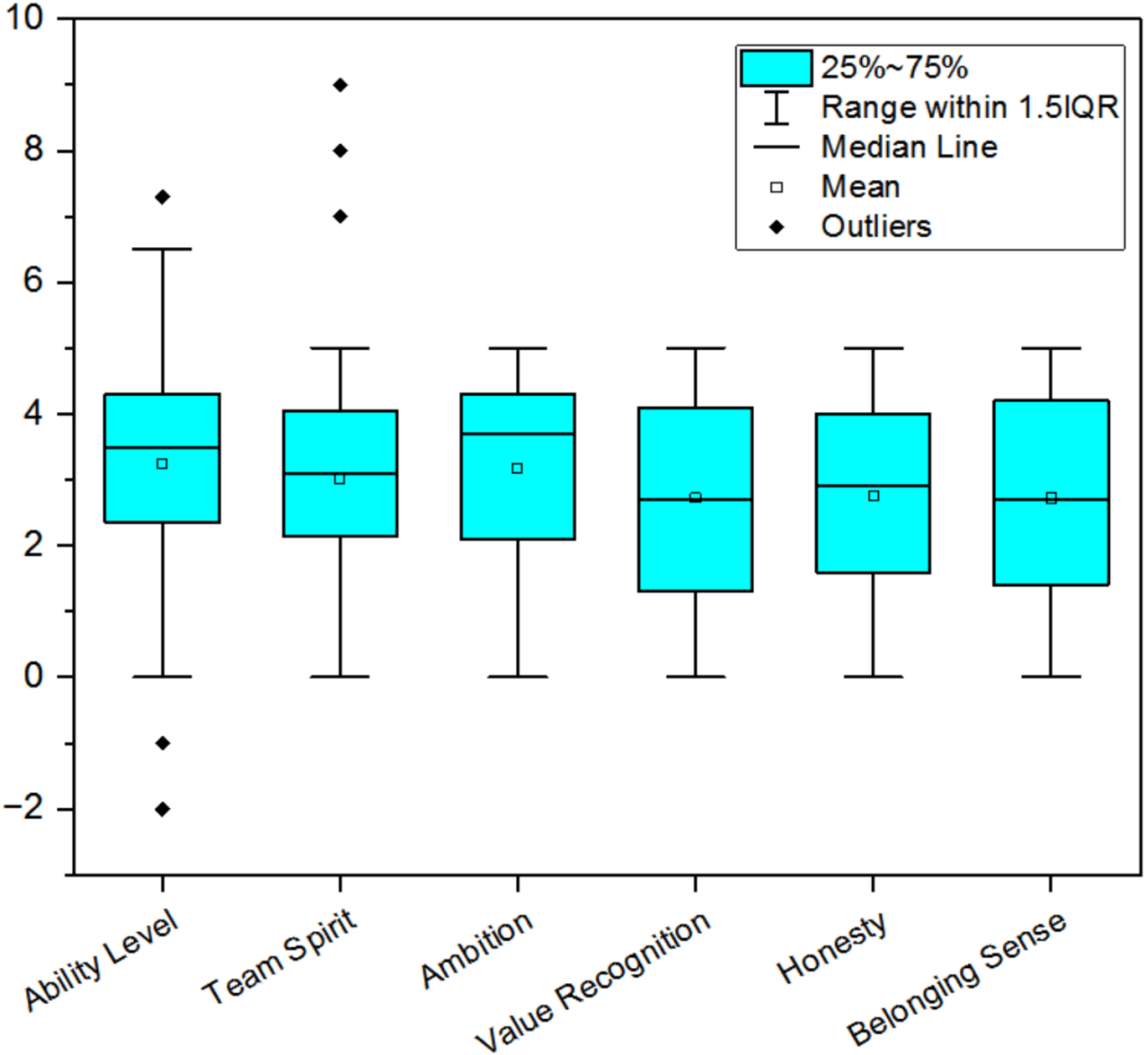
Box and Line Diagram of Abnormal Value Detection.

The visualization results are shown in Fig. 2 According to the box graph above, some variables have abnormal values, such as Ability Level variable and Team Spirit variable. As shown from the collected data set, the scale of these variables is 0–5.

Therefore, the scores below 0 and above 5 are abnormal values, indicating the need to process these variables to filter out the data within the normal range.

After the simple pre-processing of the above data, missing values, outliers and duplicate values are processed to obtain a clean and complete data set, and the data are saved to tables and databases.

Before data mining, descriptive statistics is necessary.

### Descriptive statistics

Descriptive statistics are summarized in a way that reveals the characteristics of data distribution and can be used to express quantitative data. Descriptive statistics includes data frequency analysis, data trend analysis, data dispersion analysis, distribution description and statistical graph analysis.

Table 3 carries out statistical analysis on all attributes, including both discrete data and continuous data. Some statistical indicators of discrete data are useless data, which are represented by NaN. Similarly, some statistical indicators of continuous data are unavailable, which are represented by NaN.

**Table 3:** Descriptive Statistical Analysis.

Predictors	unique	top	frep	mean	std	min	25%	50%	75%	max
ID	NaN	NaN	NaN	356.5	205.68098	1.0	178.75	356.5	543.25	712.0
Evaluation ID	667	EV6137	3	NaN	NaN	NaN	NaN	NaN	NaN	NaN
Age	NaN	NaN	NaN	35.30478	5.82647	25.0	30.0	35.0	40.0	35.0
Gender	2	Male	377	NaN	NaN	NaN	NaN	NaN	NaN	NaN
Educction	5	Doctorate	231	NaN	NaN	NaN	NaN	NaN	NaN	NaN
Position	3	medium	329	NaN	NaN	NaN	NaN	NaN	NaN	NaN
Salary	3	medium	363	NaN	NaN	NaN	NaN	NaN	NaN	NaN
Vacation	3	many	351	NaN	NaN	NaN	NaN	NaN	NaN	NaN
Welfare	3	high	349	NaN	NaN	NaN	NaN	NaN	NaN	NaN
Ability Level	NaN	NaN	NaN	3.23513	1.26974	0.0	2.4	3.5	4.268231	5.0
Team Spirit	NaN	NaN	NaN	3.01041	1.33379	0.0	2.142324	3.1	4.0	5.0
Ambition	NaN	NaN	NaN	3.17247	1.41299	0.0	2.1	3.7	4.3	5.0
Value Recognition	NaN	NaN	NaN	2.72653	1.52688	0.0	1.3	2.7	4.1	5.0
Honesty	NaN	NaN	NaN	2.7492	1.41946	0.0	1.6	2.9	4.0	5.0
Belonging Sense	NaN	NaN	NaN	2.7199	1.51914	0.0	1.4	2.7	4.2	5.0
Training Opportunities	3	high	255	NaN	NaN	NaN	NaN	NaN	NaN	NaN
Promotion Opportunities	3	medium	357	NaN	NaN	NaN	NaN	NaN	NaN	NaN
Working Environment	3	excellent	258	NaN	NaN	NaN	NaN	NaN	NaN	NaN
Overtime	3	medium	364	NaN	NaN	NaN	NaN	NaN	NaN	NaN
Label	NaN	NaN	NaN	1.438202	0.496515	0.0	0.0	0.0	1.0	1.0

As seen from the figure, descriptive statistics includes count, unique, top, frequency, mean, std, min, 25%, 50%, 75%, max and other statistical indicators related to each continuous variable.

Unique represents the unique value while top represents the attribute value with the largest frequency; frep represents the frequency value with the largest frequency; mean represents the average value of the sample, while std represents the standard deviation of the sample, and min represents the minimum value of the sample. Then, 25%, 50%, 75% are quartiles, respectively, the upper quartile, median and lower quartile, and finally, max represents the maximum value of the sample.

For example, for the discrete value of Position variable, the medium value has the largest frequency. For the continuous value of Ambition variable, the mean is 3.17, std is 1.41, min is 0; 25%, 50%, 75% is 2.1, 3.7, 4.3; max is 5.

### The distribution of characteristic data

This research analyzed several continuous characteristic variables such as Ability Level, Team Spirit, Ambition, Value Recognition, Honesty, and Belonging Sense through data visualization technology.

**Fig. 3:**
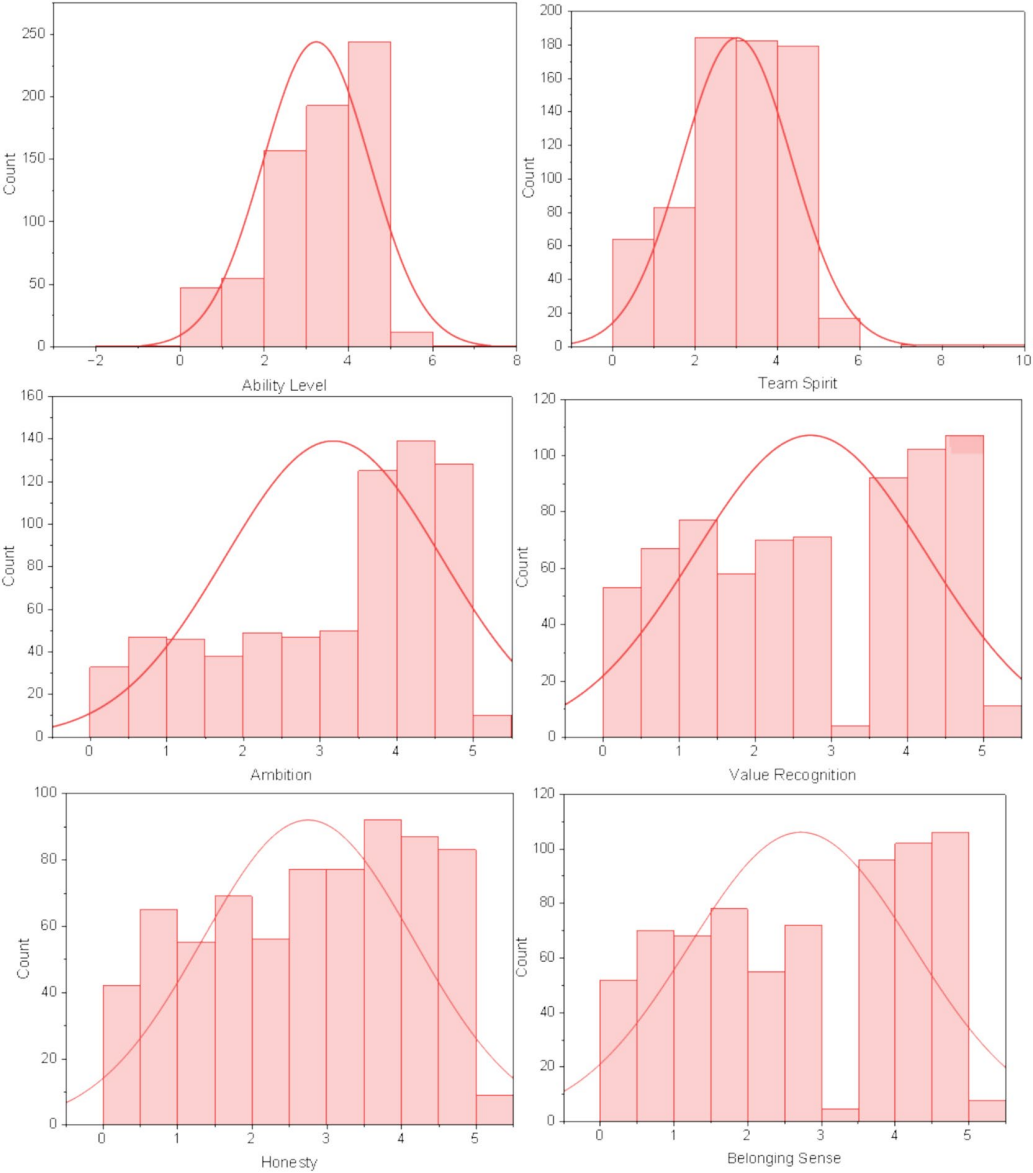
The distribution of characteristic data.

As seen from Fig. 3, the numerical distribution of each characteristic variable is irregular.

Ability Level is the evaluation of the ability level of employees. The scores are mainly 2 to 5. The highest score is 5. Among them, the highest distribution is between 4 and 4.3, indicating that the ability level of the company’s historical employees is relatively high.

Team Spirit represents the team spirit of employees, focusing on 2 to 5 points. Moreover, the number of employees with 4.7 to 5 points is the largest, indicating that there are more employees with good team spirit.

Ambition represents the level of ambition of employees. The data focus on 3.5 to 5, indicating that the number of ambitious employees in the enterprise is large.

Value Recognition represents the level of employees’ recognition of the value of the enterprise. As seen from the figure, some scores are 0 to 3, while some are 3 to 5. This further indicated that there is a polarization of employees’ Recognition of the Value of the enterprise.

Honesty represents the level of employees’ honesty. The data is evenly distributed between 0 and 5 points, and there are more employees with more than 3 points, indicating that the honesty of employees in this enterprise is generally high.

Belonging Sense represents employees’ sense of belonging to the enterprise, which is like Value Recognition. Some scores are 0 to 3, while others are 3 to 5, showing polarization.

### Character analysis

**Fig. 4:**
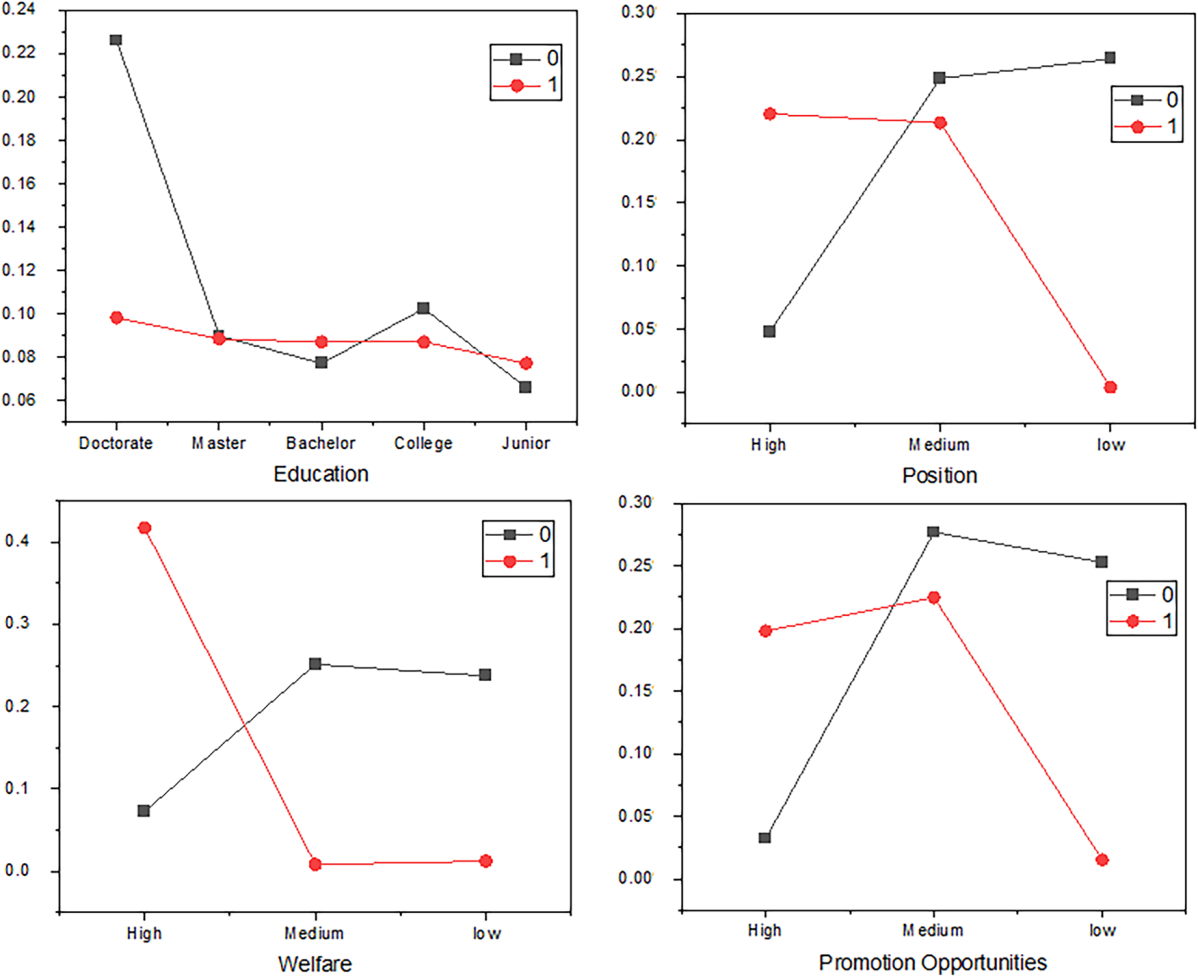
The Relationship between Features and Prediction Label.

Figure 4 shows the relationship between education and prediction labels, Position and prediction labels, Welfare and prediction labels, and Promotion Opportunities and prediction labels. Figure 4 specifically depicts the distribution of labels, so the labels are displayed in the diagram for an intuitive description.. The Label value is equal to 0, indicating that employees are disloyal or have low loyalty. However, as reflected, the label value is 1, indicating that employees are loyal or have high loyalty.

Several categorical variables related to prediction labels are analyzed.This research presents part of the process of variable analysis.

In Fig. 4, the label value is equal to 0, indicating low employee loyalty, while the value Label which is equal to 1 indicates high employee loyalty. The proportion of employees with a doctoral background marked as disloyal is 22.61%, while the proportion of employees with a doctoral background marked as loyal is 9.83%; the proportion of employees marked as disloyal with a bachelor’s degree is 7.72%, while the proportion of employees marked as loyal with a bachelor’s degree is 8.71%. In addition, as reflected in the table, the loyalty of employees with an education level of Doctorate is more likely to be low.

In Fig. 4, the proportion of employees with high positions marked as disloyal is 4.82%, while the proportion of employees with high positions marked as loyal is 22.05%; the proportion of employees marked as disloyal in low positions is 26.40, while the proportion of employees marked as loyal in low positions is 0.42%. Additionally, the probability of high loyalty is far higher than the number of employees with low loyalty when the position is high. However, the position of the lower group is the opposite, indicating that the level of loyalty is closely related to the level of the position.

In Fig. 4, the proportion of employees marked as disloyal with high benefits is 7.30%, while the proportion of employees marked as loyal with high benefits is 41.71%; the proportion of employees marked as disloyal with moderate benefits is 25.14%, while the proportion of employees marked as loyal with moderate benefits is 0.84; the proportion of employees marked as disloyal with low benefits is 23.74%, while the proportion of employees marked as loyal with low benefits is 1.26%. In addition, the welfare level is medium and low and the number of disloyal employees is more than the number of loyal employees, while for higher-level employees, the number of employees with high loyalty is more than the number of employees with low loyalty.

In Fig. 4, the proportion of employees marked as disloyal with high promotion opportunities is 3.23%, while the proportion of employees marked as loyal with high promotion opportunities is 19.80%; the proportion of employees marked as disloyal with low promotion opportunities is 25.28%, while the proportion of employees marked as loyal with low promotion opportunities is 1.54%. In addition, the promotion opportunity level is medium and low. The number of disloyal employees is more than the number of loyal employees, while at the high level, the number of employees with high loyalty is more than the number of employees with low loyalty.

### Relevant analysis

**Fig. 5:**
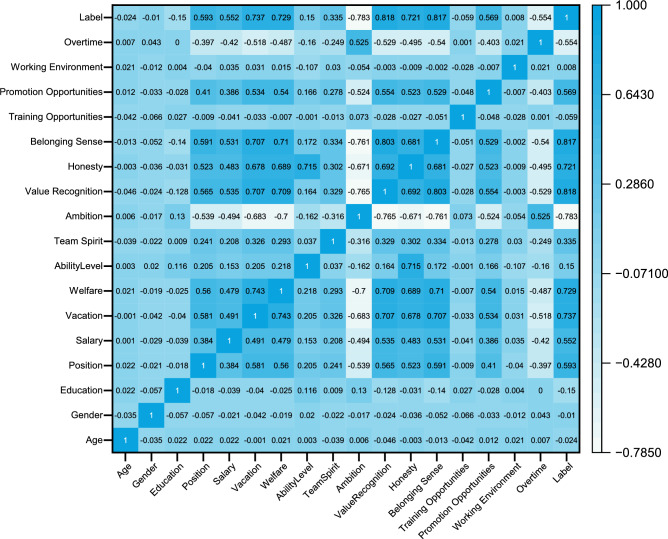
Thermodynamic Diagram of Correlation Analysis between Features.

The correlation between features is analyzed below. The correlation coefficient is visualized by the thermodynamic diagram, and the display results are shown in Fig. 5.

In the thermodynamic diagram, the lighter the color is, the stronger the positive correlation is however, the darker the color is, the stronger the negative correlation is.

It can be seen from the figure that the Loyalty Label has a strong positive correlation with Belonging Sense, Value Recognition, Honesty, and Team Spirit. The correlation coefficient with Belonging Sense and Value Recognition, indicating a strong positive correlation. This further suggests that the bigger these values are, the more loyal the employees are. However, loyalty has a strong negative correlation with the Ambition feature, reaching -0.783, indicating that the bigger the value, the smaller the loyalty.

In TSMEs, Belonging Sense is an employee’s sense of identity, security and value to a certain group or organization. Loyalty labels are a measure of how loyal an employee is. When employee feel a strong sense of belonging to a group or organization, they are more likely to show high levels of loyalty. Value Recognition is an employee’s ability to judge and evaluate the value of things or behaviors. The formation of loyalty labels is often based on the employee’s identification and acceptance of the values of the group or organization. When employees believe that the values of a group or organization are in line with their own values, they are more likely to develop a sense of loyalty to that group or organization. Loyal employee are more likely to adhere to the principle of integrity because they are more willing to be honest and trustworthy for the good of the group or organization. Team Spirit is the group spirit formed within the team, which is consistent, mutual support, close cooperation and selfless dedication. Employee with high loyalty labels tend to have stronger team spirit, and they are more willing to make efforts for the common goals of the team and maintain good cooperative relations with team members.

Feature variables “Evaluation ID” and “ID” belong to useless or meaningless feature variables. They will be removed and will not participate in machine learning training. Through the correlation degree analysis, because the relationship value of the current 17 variables is not 0, and there are not many existing feature variables, so to accurately predict, the existing 17 features are all involved in machine learning training.

### Data modeling

This step is the core of the data mining work of this research. The model construction is the generalization of the sampling data track, which reflects the general characteristics of the internal structure of the sampling data and is basically consistent with the specific structure of the sampling data. The construction of the prediction model includes model establishment, model training, model verification and model prediction. This research adopts the Python program which is more convenient for developing machine learning models. Seventeen predictors were selected to participate in machine learning training. Important predictors include Age, Gender, Education, Position, Salary, Vacation, Welfare, Ability Level, Team Spirit, Ambition, Value Recognition, Honesty, Belonging Sense, Training Opportunities, Promotion Opportunities, Working Environment, Overtime.

## Results and discussion

### Model performance comparison

In terms of prediction model selection, the main focus is on predicting employee loyalty through employee features. This is a typical classification problem. The classification algorithm is called pattern recognition and its main purpose is to discover patterns from the data and divide the data into different categories. By calculating and analyzing the training set of known classes, the classification algorithm finds the class rules and predicts the class of new data. Common classification algorithms include Decision Tree, Naive Bayes, Logistic Regression, K-nearest neighbor, Support Vector Machine and so on. Classification algorithms are widely used in finance, healthcare, e-commerce and other fields to help people better understand and use data. Cross-validation is a more rigorous method of validation that trains the model by splitting the data set into multiple parts and then taking turns using one part of it as the validation set and the rest as the training set. This approach ensures that the model is validated on different subsets of data, thus improving the accuracy and reliability of the evaluation. When the dataset contains different classes of TSMEs, cross-validation across different TSMEs classes can further ensure the generalization ability of the model across different classes, effectively reducing the risk of overfitting and improving the reliability of the model. In the following, the 8 models are trained and tested using a ten-fold cross validation method. The dataset is first divided into ten parts, then nine of them are rotated as training data and the remaining one is used as test data, and finally, the model training is performed by maximizing the use of samples by averaging the correct rate each time as the evaluation value of the algorithm accuracy.

Confusion matrix is an important tool for evaluating the performance of classification models. It clearly shows the results of the model’s classification of samples in matrix form, which helps us understand the performance of the model in different categories.

- True Positive (TP): The model correctly predicts that the sample that is actually in the positive category is in the positive category.
- False Negative (FN): The model incorrectly predicts a negative class for a sample that is actually a positive class.
- False Positive (FP): The model incorrectly predicts a positive category for a sample that is actually a negative category.
- True Negative (TN): The model correctly predicts a negative category for a sample that is actually a negative category.

**Fig. 6:**
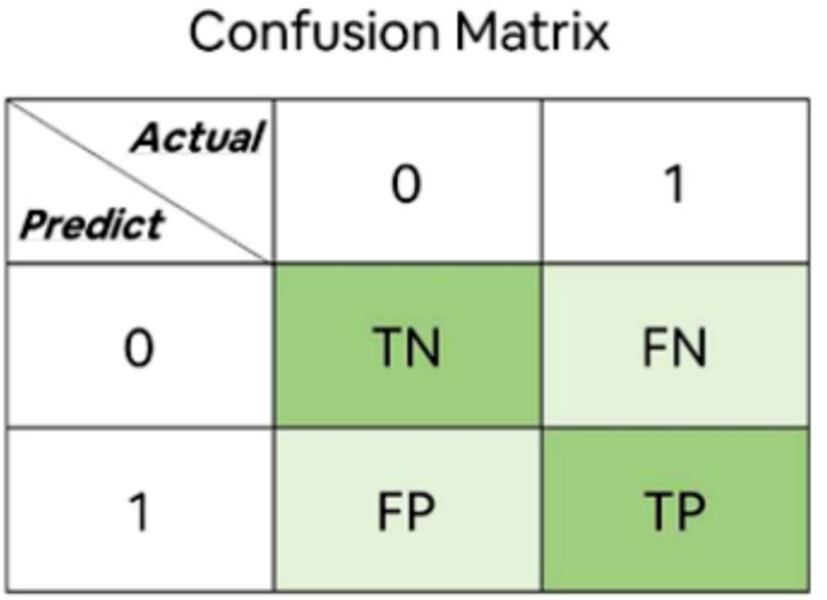
Confusion Matrix Digram.

Figure 6 shows the specific illustrations and announcements. Accuracy represents the proportion of samples correctly classified by the model to the total number of samples. Precision indicates how many of the samples that the model predicts to be positive classes are actually positive classes. Recall is the percentage of all samples that are actually positive categories that the model can correctly predict. F1-score is the harmonic average of accuracy and recall, which combines the performance of both. It’s the harmonic average of the accuracy rate and the recall rate, which combines the performance of both.

Kappa coefficient calculates the accuracy of the model prediction by comparing the positive and negative cases predicted by the model with the positive and negative cases actually classified. It takes into account not only the accuracy of the model to predict positive cases but also the accuracy of the model to predict negative cases. The higher the value of the Kappa coefficient, the higher the classification accuracy achieved by the model. The range of kappa taking values represents different degrees. 0.1∼0.2: slight; 0.2∼0.4: fair; 0.4∼0.6: moderate; 0.6∼0.8: substantial; 0.8∼1.0: almost perfect^43^.

In this research, DT, KNN, NB, LR, RF, NN, GBDT, and SVM machine learning algorithms are used successively to predict employee loyalty.

- DT: The DT algorithms predict target variables by dividing the data set into different subsets. It starts at the root node, divides the data set according to some feature, and recursively generates more child nodes until the stop condition is met. Each inner node of the decision tree represents a judgment condition on a feature attribute, each branch represents a possible attribute value, and each leaf node represents a classification result. The DT algorithm can show the decision process intuitively and is easy to understand and explain. It can recursively segment data set to form a tree structure, and can automatically process high-dimensional data without feature selection. Decision trees have high accuracy in dealing with classification problems and can deal with nonlinear relations.
- KNN: The KNN algorithm calculates the distance between the items to be classified and each item in the data set according to the distance metric, then selects the K items with the nearest distance, votes according to the categories of these K items, and takes the category with the most votes as the prediction category of the items to be classified. KNN algorithm is simple, intuitive and easy to implement. It is an instance-based learning method, which does not need to build complex models and has no strict requirements on the distribution of data. KNN algorithm performs well in multi-classification problems and is robust to unbalanced data sets.
- NB: The NB algorithm is a classification algorithm based on Bayes’ theorem that predicts target variables by calculating the conditional probability of each class. The naive Bayes algorithm assumes that each feature is independent from each other, and then calculates the conditional probability of the class based on this assumption, and selects the class with the greatest probability as the prediction result. The NB algorithm is based on Bayes theorem and has a solid mathematical foundation. It assumes that the features are independent of each other, thus simplifying the calculation process and improving the efficiency of the algorithm. The NB algorithm performs well when dealing with sparse data sets such as text classification.
- LR: The Logistic regression algorithm is an algorithm for binary classification that maps the results of linear regression to a range of 0 to 1 by using logical functions. A logistic regression model represents the relationship between input features and output categories as a linear regression function, which then converts the result of the linear regression into a probability value that can be used to predict the target variable. The Logistic regression algorithm has wide applicability and can deal with binary classification and multi-classification problems. It predicts the classification result by calculating the probability, and has the characteristic of strong interpretability. It has high accuracy when dealing with linearly separable data sets.
- RF: Based on the decision tree algorithm, the RF algorithm improves the classification accuracy by constructing multiple decision trees and combining their prediction results. It generates multiple decision trees by randomly selecting samples and features and then votes the maximum number of results as the final classification result. The RF algorithm improves the stability and accuracy of the model by constructing multiple decision trees. It can handle high-dimensional data and has a certain robustness to missing and outliers of data. The RF algorithm have significant advantages in avoiding overfitting and are able to assess the importance of variables.
- NN: The Neural network is a classification algorithm based on artificial neurons that trains the connection weights between neurons to predict target variables. A neural network consists of multiple neurons, each of which receives an input signal and generates an output signal. The output signal is multiplied by the input signal of the next neuron by the connection weight. The final output signal consists of all neurons. The Neural network has powerful nonlinear fitting ability and can deal with complex data relationship. It automatically extracts features from data through the training process, reducing the need for manual feature engineering. The Neural network perform well in processing complex data such as images and speech, and can handle multi-modal data.
- GBDT: The GBDT algorithm is a gradient-lifting based classification algorithm that predicts target variables by combining the results of multiple decision trees. The GBDT algorithm improves the accuracy of the model by adding trees, updating model parameters, and optimizing the objective function. The GBDT algorithm iteratively trains multiple weak classifiers and combines them into one strong classifier. It can handle data with both continuous and discrete values, and has no strict requirements on the distribution of data. GBDT has high accuracy in predicting continuous values and is capable of handling nonlinear relationships.
- SVM: The SVM algorithm divides different classes by finding a hyperplane. It tries to maximize the boundary between the two categories, which is called the interval. The goal of SVM is to find a hyperplane that can correctly classify the points in the data set while maximizing the spacing. In a binary classification problem, SVM finds this hyperplane by solving a quadratic optimization problem. The SVM algorithm has a solid mathematical foundation. It can handle high-dimensional data and has invariance to scaling and rotation of the data. The SVM algorithm performs well on both linearly separable and nonlinearly separable data sets, and can handle nonlinear relationships through kernel functions.

**Fig. 7:**
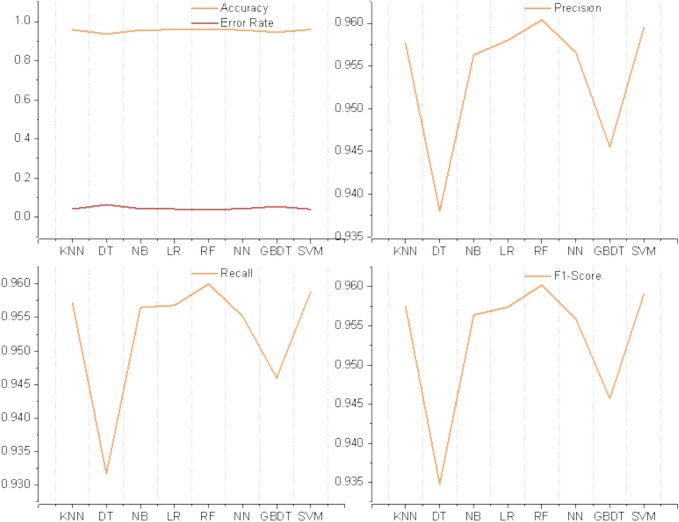
Performance value comparison of multiple model prediction results.

The above 8 algorithms are popular and concerned with predictive modeling supervision algorithms for data mining and machine learning. The optimal prediction model is selected by using the grid search method for hyperparameter debugging. After the parameter tuning of each model, the prediction results of each model are obtained as shown in Table 4. The model performance was compared by calculating Accuracy, Precision, Recall, Error Rate, and Kapaa. The visual illustration of the prediction models is shown in Fig. 7.

**Table 4:** Prediction results of the classification model.

Model	Accuracy	Precision	Recall	F1-Score	Error Rate	Kapaa
DT	93.55%	93.80%	93.17%	93.48%	6.45%	0.868
KNN	95.79%	95.76%	95.72%	95.74%	4.21%	0.914
NB	95.65%	95.63%	95.65%	95.64%	4.35%	0.912
LR	95.79%	95.80%	95.68%	95.74%	4.21%	0.914
RF	96.07%	96.04%	96%	96.02%	3.93%	0.920
NN	95.65%	95.66%	95.52%	95.59%	4.35%	0.912
GBDT	94.52%	94.55%	94.60%	94.57%	5.48%	0.889
SVM	95.92%	95.95%	95.88%	95.91%	4.08%	0.917

From the analysis of the prediction results, the highest Accuracy is RF, reaching 96.07%, followed by SVM, KNN, LR, NN, NB, GBDT, DT; the highest Precision is RF, reaching 96.04%, followed by SVM, LR, KNN, NN, NB, GBDT, DT; the highest Recall is RF, reaching 96%, followed by SVM, KNN, LR, NB, NN, GBDT, DT; the highest F1-Score is Random Forest, reaching 96.02%, followed by SVM, KNN, LR, NB, NN, GBDT, DT. By comparison, RF has the highest scores in prediction Accuracy, Precision, Recall and F1.

**Fig. 8:**
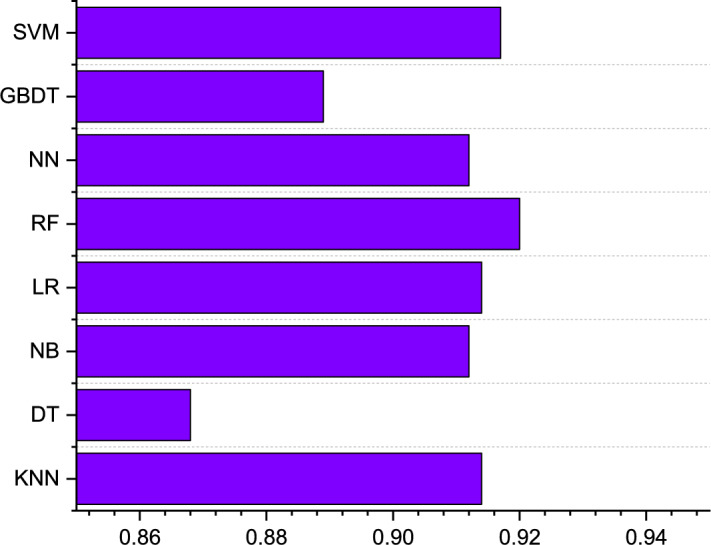
Comparison of Kapaa results from multiple models.

**Fig. 9:**
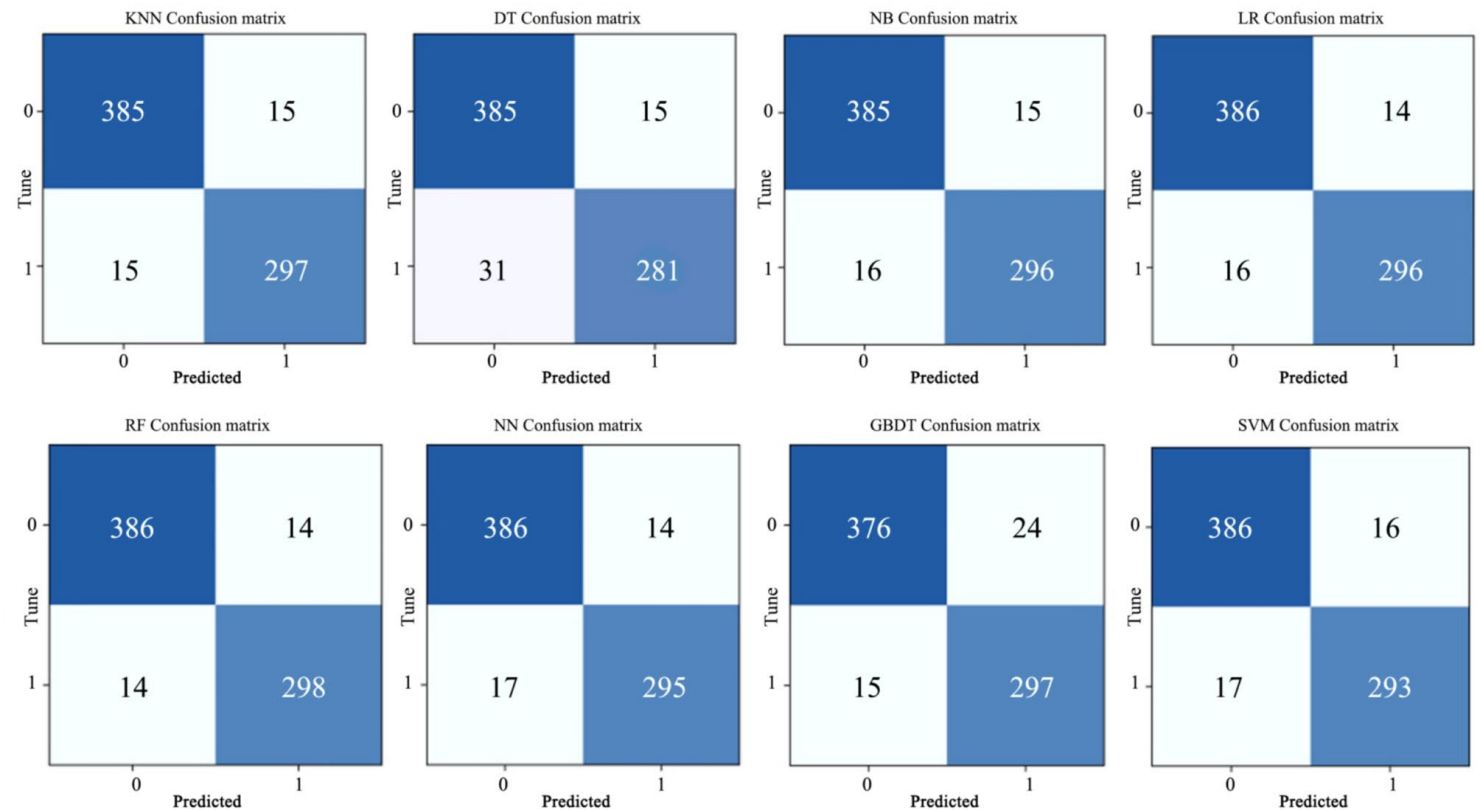
Confusion matrix for different models.

The kappa of each model kappa indicates that the overall consistency and classification consistency of each model are normal and meet the requirements. Figure 8 shows a comparison of Kapaa results for multiple models. The highest Kapaa is RF, reaching 0.920, followed by SVM, KNN,LR,NN,NB,GBDT,DT. The confusion matrix visualizes the relationship between the predictions of the classification model and the actual labels. Figure 9 shows the confusion matrix diagram of the different models. These results demonstrate the effectiveness of the RF model to accurately predict employee loyalty in TSMEs under comprehensive conditions. The results of all indicators show that the RF prediction model has the best performance and can be used to Predict employee loyalty in TSMEs. Through comparative analysis of other studies, machine learning shows satisfactory results in predicting different dimensions of employees, but no machine learning algorithm has been found to consistently perform the best in predicting different dimensions of employees^16,18,28–30,38^.

### Exploratory analysis

In RF models, feature importance assessment is a key task that helps us understand how much each feature contributes to classification accuracy^44^. Feature importance provides a way to effectively quantify the ability of input features to predict model outputs. In a random forest, the higher the importance score of a feature, the greater the role that feature plays in the model prediction. This is useful for understanding data, making feature selections, and interpreting model predictions. The following is a detailed and practical exploration of the importance of features.

The importance of the feature is measured by calculating the average information gain generated by the feature in the decision-making process of each decision tree. The importance of each feature is obtained by using the “feature_importances_ attribute”. The total importance of all features is 1.0.

**Fig. 10:**
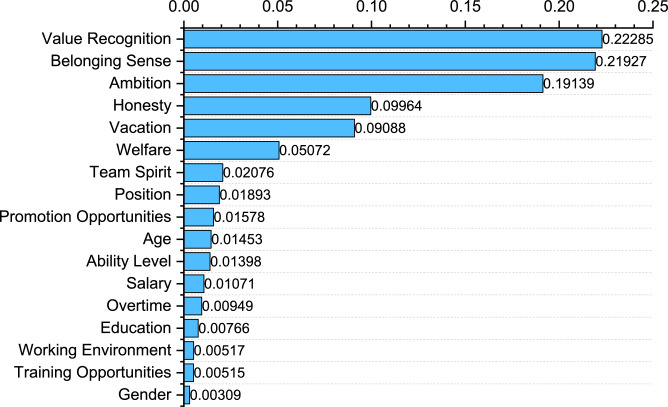
Characteristic Importance of Impact Prediction Variables.

As can be seen from Fig. 10: The proportion of Value Recognition is 0.22285, which has the highest weight and plays a key role in model construction. The proportion of Belonging Sense is 0.21927, which is the second most important feature and plays an important role in model construction. Ambition accounted for 0.19139; Honesty was 0.09964%; The proportion of Vacation is 0.09088%; The proportion of the above 5 features accounted for 0.82403; The remaining 12 items include Welfare, Team Spirit, Position, Promotion Opportunities, Age, Ability Level, Salary, Overtime, Education and Working The proportion of Environment, Training Opportunities and Gender is as follows: 0.05072, 0.02076, 0.01893, 0.01578, 0.01453, 0.01398, 0.01071, 0.00949, 0.00766, 0.00517, 0.00515, 0.00309.

**Fig. 11.**
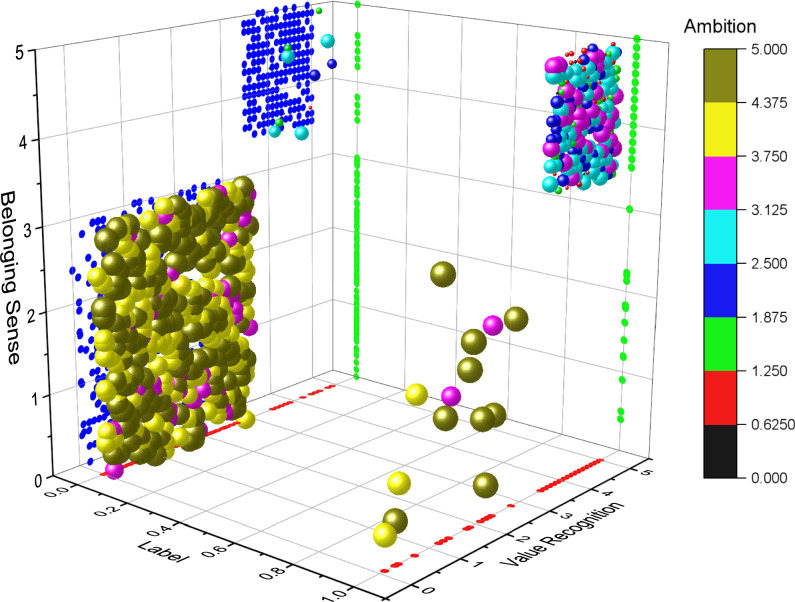
3D scatterplot of important features.

Scatterplot refers to the distribution of data points on the cartesian coordinate plane in regression analysis. The scatterplot represents the approximate tendency of the dependent variable to change with the independent variable, according to which the appropriate logarithm of function data points can be selected for fitting. The scatterplot matrix is used to draw the scatterplot between the respective variables at the same time so that the main correlation between multiple variables can be quickly discovered. Targeted feature selection was carried out on the data set, and a multivariate 3D scatter plot was drawn. The X-axis was Label, the Y-axis was Value Recognition, the Z-axis was Belonging Sense, and Ambition was color mapping. It can be intuitively concluded from Fig. 11 that when the lable is 1, the larger the Value of Value Recognition and Belonging Sense, the closer the Ambition color is to the upper part of the color band. When the lable is 1, the smaller the Value of Value Recognition and Belonging Sense, the closer the Ambition color is to the lower part of the color band. The diagram shows that the analysis results are relatively concentrated, which further confirms the content of the relationship analysis discussed above.

Based on the above research findings and interviews with enterprises, it is necessary for enterprises to make efforts at multiple levels to enhance employee loyalty. This study puts forward the following suggestions and measures.

Clarifying and strengthening enterprise values is the foundation of building an efficient corporate environment. When enterprise values are closely aligned with its mission, vision and goals, it can form a strong internal cohesion that guides employee behavior and decision making. Value co-creation plays an obvious role in the sustainable development of enterprises^45^.This consistency not only helps shape a company’s unique culture, but also enhances employee belonging and loyalty. In practical terms, this means that enterprise need to integrate these values into daily operations, recruitment, training, assessment and other aspects to ensure that all employees understand and practice these values. However, translating values into practical action is not easy. On the one hand, companies need to ensure that their values are articulated clearly and easily understood, avoiding vague or overly abstract expressions. On the other hand, senior managers and leaders must lead by example and embody these values through their actions and decisions. In addition, TSMEs also need to establish an effective feedback mechanism to timely understand the understanding and acceptance of the values of employees in order to adjust and optimize.

How to retain the core talents, mobilize their enthusiasm, and give full play to their potential requires the reform and management of enterprises^46^. Relevant research results also show that talent management is an important predictor of competitive advantage^47^. According to the characteristics of "specialized, refined, special and new" of TSMEs, it is an important means to develop personalized talent training programs to enhance the competitiveness of enterprises. By strengthening talent training and support, enterprises can help employees improve their skills and knowledge level, achieve personal career goals, and provide a continuous talent reserve for the development of enterprises. This investment strategy can not only enhance employee satisfaction and loyalty, but also enhance the innovation ability and market adaptability of the enterprise. Talent development investment requires enterprises to invest a lot of resources and time. In addition, how to ensure that the training content matches the actual needs of the enterprise, how to evaluate the training effect and continuously optimize the training plan are also challenges that the enterprise needs to face. Therefore, enterprises need to formulate a scientific training budget and plan, choose the appropriate training methods and contents, and establish an effective evaluation mechanism to track the training effect.

A positive, inclusive and supportive work environment is one of the key factors in attracting and retaining talent. Corporate culture is closely related to employee enthusiasm and employee performance^48^. By strengthening the construction of corporate culture, TSMEs can create an atmosphere that makes employees feel comfortable and respected, thus improving the enthusiasm and creativity of employees. At the same time, being transparent and communicating honestly with employees about current situations, challenges, and plans helps build relationships of trust and enhances employees’ sense of belonging and responsibility. Enterprise culture construction is a long and complicated process. Companies need to invest a lot of time and energy in shaping and maintaining corporate culture. In addition, different employees may have different levels of understanding and acceptance of culture, which may lead to cultural conflicts and misunderstandings. Therefore, enterprises need to formulate clear cultural construction goals and plans, promote and strengthen corporate culture through training, publicity, activities and other ways, and establish an effective communication mechanism to solve cultural conflicts and misunderstandings.

Establishing a reward and commendation system to recognize and motivate employees who embody corporate values in their work is an effective means to stimulate employees’ work enthusiasm and creativity. By encouraging the incentive of research and development and highly skilled talents, TSMEs can stimulate the innovative spirit and entrepreneurial enthusiasm of talents, thereby promoting the sustainable development and innovation of enterprises. This incentive mechanism can not only improve the satisfaction and loyalty of employees, but also enhance the cohesion and competitiveness of enterprises. Research has shown that corporate ESG behavior has the potential to enhance employee loyalty and satisfaction. Employees who support ESG attitudes are more likely to recognize and endorse corporate ESG behavior^49^. Developing and implementing effective incentive mechanisms requires enterprises to consider several factors. On the one hand, enterprises need to ensure the sustainability of the incentive mechanism, and avoid over-reliance on material rewards that lead to utilitarian mentality of employees. On the other hand, enterprises also need to consider the needs and expectations of different employees, and formulate personalized incentive programs to meet their different needs.

It is of great significance for the government to strengthen the public service function to build an efficient enterprise environment and promote the training of scientific and technological innovation talents^50,51^. Improving the public personnel service network system at all levels can provide comprehensive and convenient talent information services, talent integrity services, professional ability evaluation services and human resources services for TSMEs. These services help enterprises to reduce recruitment costs, improve recruitment efficiency, optimize talent structure, and thus enhance the competitiveness of enterprises. At the same time, the government can also provide a good policy environment for the training of scientific and technological innovation talents by formulating relevant policies and measures, such as tax incentives and talent introduction. These policies and measures can stimulate the innovation vitality of enterprises, attract and retain outstanding talents, and promote scientific and technological innovation and industrial upgrading. As enterprises in different regions and industries may have different needs for public services, the government needs to formulate differentiated policy measures to meet these needs. How to ensure the continuity and stability of public services is also a challenge. The government needs to establish a long-term mechanism and system to ensure the continuous provision and continuous optimization of public services.

To sum up, building an efficient enterprise environment requires enterprises to make efforts in clarifying values, investing in talent development, building corporate culture and employee recognition and incentive, and seeking government support strategies.

## Conclusions

The proposed machine learning model is evaluated by using confusion matrix and model performance score. RF prediction model has the best comprehensive performance. DT, KNN, NB, LR, RF, NN, GBDT, and SVM also show good prediction results. This shows that it is possible to predict the employee loyalty of TSMEs through machine learning classification algorithms. The existing research on employee loyalty prediction is limited. Comparing current research data, including employee promotion and loyalty prediction based on improved AdaBoost machine learning methods, logical impact research on employee management and loyalty based on machine learning, and use of machine learning techniques to predict job satisfaction and employee behavior, the random forest analysis method proposed in this paper is superior to all other methods in predicting employee loyalty in technology-based smes, with an accuracy of 96.07%^52–54^. It should be noted that this study is to provide decision support reference for enterprise management, and enterprises should not only rely on machine learning for processing.

The machine learning algorithm is used to mine and analyze the employee management data of the TSMEs, and determine the important characteristics and important influencing factors of the model. Several characteristics were analyzed in relation to the Prediction Label. For example, employees with education level of doctorate are more likely to have low loyalty. Relevant Analysis is carried out to analyze the degree of association between the Label and the variables by constructing a thermodynamic diagram, and to determine whether the association is positive or negative. This is of great relevance to the analysis of employee variables in order to explore the potential relationships between Labels and variables. Through correlation analysis, it is concluded that employee loyalty has a positive relationship with Value Recognition variable, Honesty variable, Belonging Sense variable and Team Spirit variable. However, it has a negative correlation with Ambition variable.

According to the importance ranking of random forest features, Value Recognition has the highest proportion and the highest contribution in model construction. It was followed by Belonging Sense, Ambition, Honesty and Vacation. According to the analysis results of characteristic factors, TSMEs should focus on the Value Recognition, Belonging Sense, Ambition, Honesty and Vacation of employees, which plays an important role in improving employee loyalty. For example, improving the Value Recognition and Belonging Sense of employees with high Ambition value can effectively enhance the loyalty of employees.

This study started in June 2023 and ended in May 2024. At present, the research on the prediction and evaluation of enterprise employee loyalty mainly adopts the methods of questionnaire survey, interview and data analysis. By collecting and analyzing the data of employees’ work attitude, work performance and turnover intention, the prediction model is built to predict enterprise employee loyalty. Aiming at TSMEs, this study provides more effective, clearer and more intelligent decision support for smes through machine learning algorithms.

This study has some limitations. Data collection and processing is an important challenge. Since employee loyalty involves many factors, a large amount of data needs to be collected for analysis. However, the collection and processing of data can be affected by a variety of factors, such as data quality, data privacy, enterprise differentiation and other factors. In addition, the accuracy and reliability of predictive models has always been a concern. Since the change of employee loyalty is affected by many factors, the prediction model should be able to accurately capture the change trend of these factors and provide reliable prediction results for evaluation. The future research direction is to strengthen the construction of employee loyalty and employee value evaluation system in TSMEs, reduce the influence of factors such as industry, difference and particularity, to achieve comprehensive data collection, real-time analysis, dynamic tracking, and ensure the systematic, scientific, standardized and long-term research.

.

## Data Availability

All relevant data are within the paper. Any interested researcher can contact author Yong Shi via email to obtain the original dataset.
